# Surgery for patients with facial palsy in Germany: a diagnosis-related-groups-based nationwide analysis, 2005–2019

**DOI:** 10.1007/s00405-023-08259-4

**Published:** 2023-09-27

**Authors:** Susanna Seeberger, Peter Schlattmann, Orlando Guntinas-Lichius

**Affiliations:** 1 Department of Otorhinolaryngology, Jena University Hospital, Am Klinikum 1, 07747 Jena, Germany; 2 Department of Medical Statistics, Computer Sciences and Data Sciences, Jena University Hospital, 07747 Jena, Germany; 3 Facial-Nerve-Center, Jena University Hospital, 07747 Jena, Germany; 4 Center for Rare Diseases, Jena University Hospital, 07747 Jena, Germany

**Keywords:** Facial paralysis, Surgery, Reanimation, Nerve reconstruction, Muscle transfer, nationwide

## Abstract

**Purpose:**

Chronic flaccid paralysis of the facial nerve leads to permanent dysfunction of eye closure, problems with drinking and eating, and lack of emotional expression. Modern facial surgery can help those affected. An analysis of the development of facial surgery in Germany over time is presented.

**Methods:**

Nation-wide population-baes diagnosis-related case group (DRG) data of virtually all inpatients who underwent facial surgery for facial palsy between 2005 and 2019 were included. Binomial regression models for changes in surgery rates over time were calculated in relation to gender and treating specialty.

**Results:**

Between 2005 and 2019, there were 28,622 inpatient stays for facial surgery. Most surgeries were provided by otolaryngology (39%) and ophthalmology or dentistry, oral and maxillofacial surgery (20% each). The mean treatment rate was 2.33 ± 0.53 surgeries per 100,000 person-years. The surgery rate was highest for nerve reconstruction surgery (0.46 ± 0.15) and static sling surgery (0.44 ± 0.0.16). The greatest increase was seen in men for nerve surgery (3.9-fold; relative risk [RR] = 3.68; confidence interval [CI] = 3.18–4.26) and sling surgery (5.0-fold; RR = 4.25; CI = 3.38–5.33).

**Conclusions:**

While nerve and sling surgery increased significantly over time, this was less true or not true at all for surgical techniques. Surgical rates and their change over time were greater in men, without explanation from the data.

## Introduction

Peripheral facial palsy is the most common cranial nerve disorder, with an estimated incidence of 7–40 patients/100,000 population per year [1]. The most common cause is idiopathic facial nerve palsy in 60–75% of cases. Trauma, inflammation, tumors or metabolic diseases are the most important other causes. Depending on the severity of the lesion, there may be complete recovery, recovery with defect healing, or failure to recover. For example, if infiltrated extratemporal nerve portions are removed during surgery of a malignant parotid tumor, or if transection of the nerve occurs at the cerebellopontine angle during surgery for a vestibular schwannoma, the patient initially develops acute paralysis. Nonetheless, in clinical practice and publications, a distinction is often not made between facial paresis and facial paralysis, instead the term palsy is used. Because of the lack of regeneration of the nerve fibers, chronic paralysis develops. In addition to complete facial paralysis, this is characterized by loss of tone with lagophthalmos, eyebrow ptosis, and drooping corner of the mouth. [2]. This leads to drinking and eating problems, poorer pronunciation, limited vision in the affected eye, and most importantly, lack of emotional expression and limitations in nonverbal communication [3, 4]. All this and the stigmatization lead to a significantly reduced quality of life, psychosocial withdrawal and often also to depression in the affected persons. [5].

Important goals of surgical therapy for patients with chronic peripheral facial paralysis (rarely, patients with central facial paresis are treated surgically) are to improve a) facial tone at rest and thus symmetry of facial expression at rest and mouth closure during drinking and eating, b) eye closure to prevent damage to the eye and maintain good vision, and c) lifting of the corner of the mouth to restore a more symmetrical smile. These measures, in turn, are intended to improve the overall quality of life of those affected. The best treatment results can be achieved with dynamic procedures when the facial nerve itself can be restored, because after reinnervation, the original facial musculature with its diverse expression patterns is reanimated again [2, 6]. If the nerve itself cannot be restored, muscle transfer or static sling surgery can be considered. With a muscle transfer, selected movements can be reconstructed. Static procedures provide better symmetry of the face at rest but do not produce motion. In addition, other procedures are often performed, for example, around the eye to improve eye closure. A number of procedures have been standardized and established internationally in recent decades [7]. Optimal surgical treatment requires expertise from different disciplines. Having a large repertoire of surgical procedures is necessary to be able to select the optimal combination of surgical procedures for the individual patient.

The aim of the present study was to provide the first nationwide inventory of inpatient surgical treatment for facial palsy in surgical rates (per 100,000 person-years) in order to obtain an overview of the surgical procedures used, the disciplines involved, and changes over the period from 2005 to 2019.

## Material and methods

### Data source

Inpatient treatment data for 2005 to 2019 were provided by the Federal Statistical Office (https://www.destatis.de/). Using the data structure files provided by the Federal Statistical Office, programs were written for SAS statistical software version 9.4 (SAS Institute Inc., Cary, North Carolina USA). After the programs were run over the original data using controlled remote data processing by the Federal Statistical Office, the anonymized results were made available for analysis after confidentiality checks. Anonymization of data is regulated by §16 of the Federal Statistics Act. If subgroups contained < 5 patients, these data were censored for data protection reasons to safely preserve the anonymity of individual cases. Since only anonymized data were analyzed, approval for data analysis by the local ethics committee was not required.

### Selection of patients and procedures of inpatient treatment

All patients with the following codes of the 10th version of the International Statistical Classification of Diseases and Related Health Problems (ICD-10-GM) were initially included: G51, G51.0, G51.1, G51.2, G51.8, and G51.9. Because of low case numbers in the subgroups for all other ICD codes, the analysis was then performed exclusively with G51.0. These cases were grouped according to the operation and procedure codes (OPS) coded for them: (a) nerve reconstruction surgery (5–04, 5–05), dynamic muscle transfer (5–853, 5–858); (b) static measures – sling surgery (5–852, 5–854, 5–855, 5–856), upper eyelid weight (5–0990); and (c) adjunctive measures—tarsorrhaphy (5–092), blepharoplasty (5–097), other eyelid reconstruction (5–096), lip and mouth corner plastic surgery (5–908), and rhytidectomy/facelift surgery (5–910). When 5-digit OPS codes were chosen for selection, this often resulted in too few cases per group, thus blocking the analysis and making it impossible to analyze. Other variables queried were: Gender, age groups in years (not shown here), state of treatment (not shown here), and selected specialty departments (neurosurgery; plastic surgery; otolaryngology; ophthalmology; dentistry, oral and maxillofacial surgery). For other specialties, case numbers were too low for analysis over the years.

### Calculation of the operation rates

The German population from 2005 to 2019 was used to calculate the surgery rates. The population data were also retrieved from the website of the German Federal Statistical Office (https://www-genesis.destatis.de/genesis/online). The cut-off date in each case was December 31 of the corresponding year. Surgery rates were related to 100,000 persons: surgery rate = (number of surgeries/population) × 100,000. Changes in surgery rates between the first year considered, 2005, and the most recent year, 2019, were calculated as follows: ((2019 surgery rate × 2005 treatment rate) / 2005 surgery rate) × 100.

### Statistical analysis

The SAS version 9.4 program (SAS Institute, Cary, NC, USA) was used for all calculations. Negative binomial regression models with log link function were calculated to perform analysis over time. Here, the dependent variable was the number of cases and the logarithm of the population at risk was used as the so-called offset. The elapsed time since 2005 was used as a covariate. Models were used to calculate the relative risk (RR) with 95% confidence intervals (CI) for change over time in each case. For all statistical tests, the significance level was two-sided and set at p < 0.05.

## Results

### Frequency of inpatient surgeries for facial palsy 2005–2019

An overview of case numbers and surgery rates is provided in Table 1. A total of 28,622 inpatient stays for facial surgeries were included in the gender comparison between 2005 and 2019. The mean treatment rate (incidence) over the 16 years considered was 2.33 ± 0.53 surgeries per 100,000 person-years. Mean surgery rates for the various surgical techniques varied from 0.17 to 0.49 in men and from 0.14 to 0.42 in women, and surgery rates were greater in men than in women for all surgical techniques considered (ratios ranging from 1.1 to 1.4). The greatest surgical rates were seen for nerve surgery (men: 0.49 ± 0.19; women: 0.42 ± 0.11) followed by sling surgery (men: 0.48 ± 0.19; women: 0.41 ± 0.13). Tarsorrhaphy followed in third place as a measure on the eye (men: 0.34 ± 0.05; women: 0.24 ± 0.03). In men, upper eyelid weighting (0.29 ± 0.08) followed as another measure on the eye, and in women, blepharoplasty (0.23 ± 0.04). Lower surgery rates were observed for reconstructions on the eyelids, reconstructions on the lip and mouth and for rhytidectomy.

**Table 1:** Frequency of inpatient facial surgery for facial palsy 2005–2019

Surgical technique	Sex	Total number of cases	n/100000/year M ± SD	Ratio 2019/2015	n/100000 Ratio m/f
Nerve surgery	m	2988	0.49 ± 0.19	3.9	1.2
	f	2612	0.42 ± 0.11	2.3	
Muscle transfer	m	909	0.15 ± 0.06	3.9	1.3
	f	774	0.12 ± 0.04	2.3	
Sling surgery	m	2872	0.48 ± 0.19	5.0	1.2
	f	2533	0.41 ± 0.13	3.0	
Lid weight	m	1741	0.29 ± 0.08	3.0	1.3
	f	1373	0.22 ± 0.05	2.1	
Tarsorrhaphy	m	2052	0.34 ± 0.05	1.3	1.4
	f	1523	0.24 ± 0.03	1.1	
Blepharoplasty	m	1560	0.26 ± 0.06	2.0	1.1
	f	1441	0.23 ± 0.04	1.3	
Eyelid surgery	m	1263	0.21 ± 0.05	2.0	1.2
	f	1094	0.18 ± 0.03	1.6	
Lip/mouth surgery	m	1007	0.17 ± 0.04	1.9	1.2
	f	851	0.14 ± 0.02	1.1	
Rhytidectomy	m	1003	0.17 ± 0.03	1.6	1.1
	f	1023	0.16 ± 0.02	1.1	
Total	m	15,398	0.28 ± 0.13	–	1.2
	f	13,224	0.23 ± 0.11	–	

*M* mean, *SD* standard deviation, *m* male, *f* female

### Changes in surgery rates for facial palsy over time from 2005 to 2019 for the different surgical techniques

**Fig. 1:**
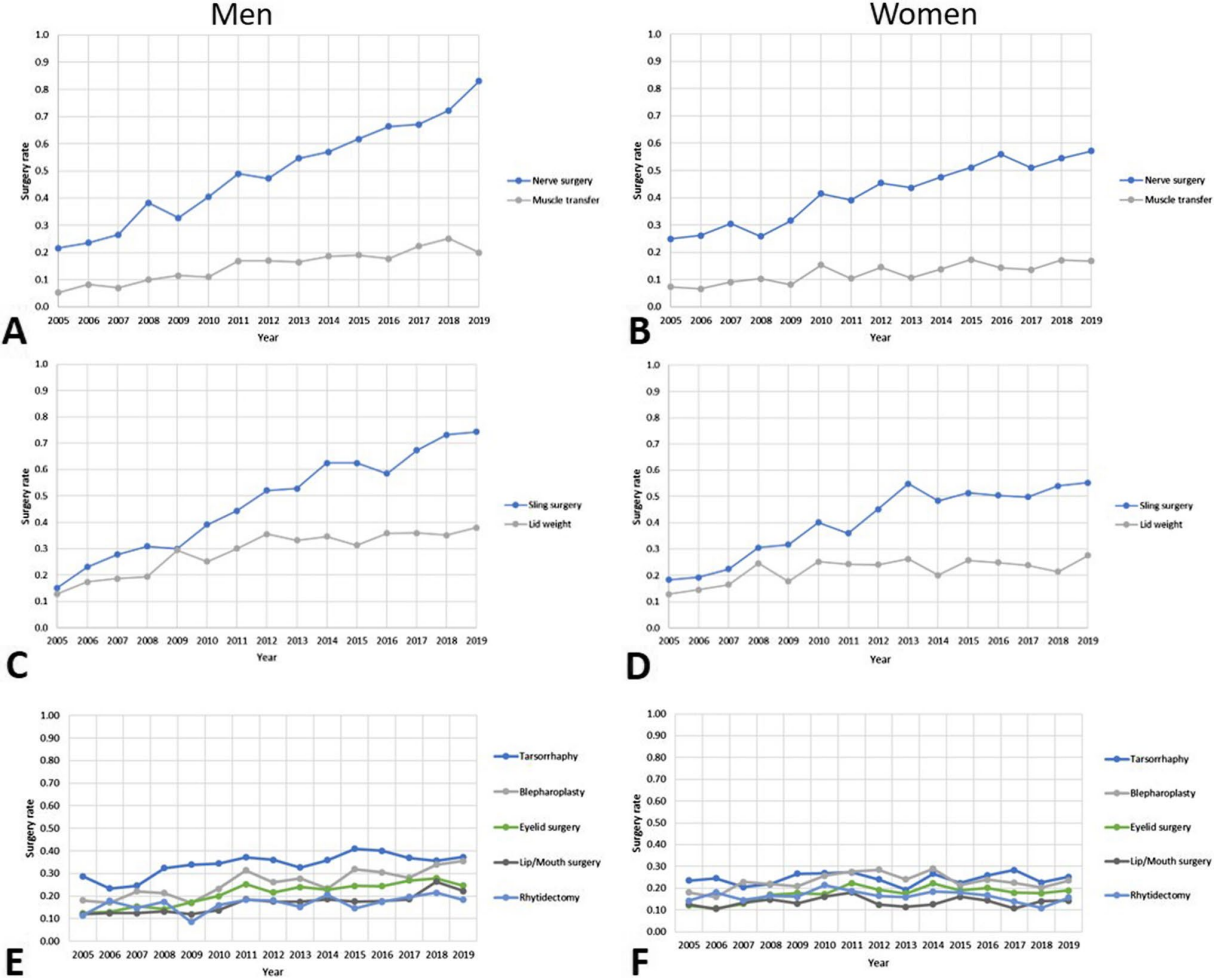
Changes in facial surgery rates per 100,000 persons over the period 2005 to 2019 for men (**A**, **C**, **E**) and women (**B**, **D**, **F**) separately for dynamic reconstruction (**A**, **B**), static reconstruction, and adjuvant procedures (**E**, **F**)

Changes in surgery rates over the years from 2005 to 2019 are shown in Fig. 1 and in Table 2. The greatest increase was seen in men for nerve surgery (increase 2019 to 2015: 3.9-fold; RR = 3.68; CI = 3.18–4.26) and sling surgery (increase 2019 to 2015: 5.0-fold; RR = 4.25; CI = 3.38–5.33). For these two surgical methods, an increase was also seen in women (increase 2019 to 2015; for nerve surgery: 2.3-fold; RR = 2.48; CI = 2.13–2.90; for sling surgery: 3.0-fold; RR = 3.12; CI = 2.32–4.11), but less steadily, especially in the last 10 years. In both sexes, a small increase over the years was seen for dynamic muscle transfer (men: 3.9-fold; RR = 3.74; CI = 2.79–5.00; women: 2.3-fold; RR = 2.35; CI = 1.76–3.13) and upper eyelid weight bearing (men: 3.0-fold; RR = 2.52; CI = 1.93–3.27; women: 2.1-fold; RR = 1.67; CI = 1.25–2.22) as a static measure on the eye. Surgery rates for tarsorrhaphy were higher than for upper eyelid weight loading, but the rate of increase was lower for tarsorrhaphy as another upper eyelid correction measure over the years and was not significantly changed in women (men: 1.3-fold; RR = 1.48; CI = 1.25–2.20; women: RR = 1.10; KI = 1.08; KI = 0.90–1.29). Similarly, when blepharoplasty was used, a significant change was seen only in men (men: RR = 2.01; CI = 1.64–2.46; women: RR = 1.20; CI = 0.92–1.56). For facelifts, there was only a slight increase in surgery rates in men (RR = 1.47; CI = 1.08–2.00) and even a trend toward a decrease in women (RR = 0.88; CI = 0.68–1.13).

**Table 2:** Changes in facial surgery rates for facial palsy over time from 2005 to 2019 for the different surgical techniques

Surgical technique	Sex	Estimate	StdErr	p-value	RR	u. 95%-CI	l. 95%-CI
Nerve surgery	m	0.0869	0.0050	< 0.0001	3.6796	3.1808	4.2567
	f	0.0607	0.0052	< 0.0001	2.4840	2.1312	2.8952
Muscle transfer	m	0.0879	0.0099	< 0.0001	3.7365	2.7931	4.9985
	f	0.0569	0.0098	< 0.0001	2.3484	1.7616	3.1306
Sling surgery	m	0.0964	0.0078	< 0.0001	4.2468	3.3815	5.3335
	f	0.0760	0.0093	< 0.0001	3.1247	2.3742	4.1126
Lid weight	m	0.0615	0.0089	0.0004	2.5162	1.9383	3.2665
	f	0.0338	0.0096	< 0.0001	1.6609	1.2521	2.2032
Tarsorrhaphy	m	0.0261	0.0058	< 0.0001	1.4790	1.2492	1.7531
	f	0.0050	0.0062	0.4193	1.0780	0.8984	1.2933
Blepharoplasty	m	0.0464	0.0069	< 0.0001	2.0045	1.6360	2.4558
	f	0.0120	0.0089	0.1785	1.1971	0.9211	1.5556
Eyelid surgery	m	0.0517	0.0068	< 0.00001	2.1724	1.7774	2.6551
	f	0.0288	0.0090	0.0014	1.5411	1.1822	2.0090
Lip/mouth surgery	m	0.0497	0.0074	< 0.00001	2.1086	1.6977	2.6189
	f	0.0036	0.0088	0.6783	1.0562	0.8157	1.3678
Rhytidectomy	m	0.0256	0.0104	0.0142	1.4688	1.0804	1.9970
	f	− 0.0087	0.0086	0.3127	0.8783	0.6827	1.1299

*Estimate* estimate of the effect of time, *StdErr* standard error of the estimator, *RR* relative risk, *l. 95% CI* lower limit of the 95% confidence interval, *o. 95% CI* upper limit of the 95% confidence interval

### Changes in surgery rates for facial palsy over time from 2005 to 2019 in the various disciplines involved

**Fig. 2:**
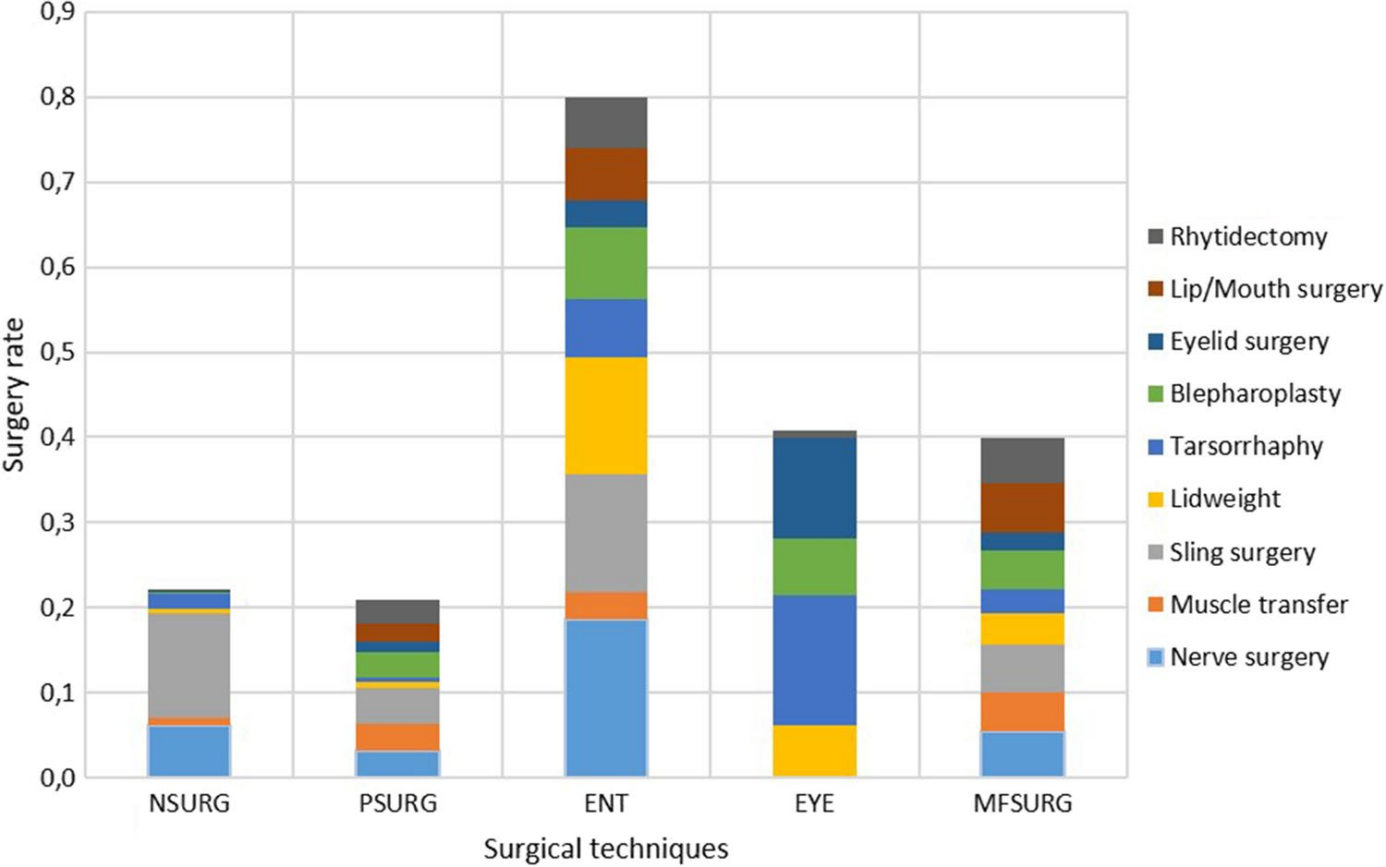
Participation of the different specialties in the average surgery rates per 100,000 persons from 2005 to 2019. The different surgical techniques are color-coded. *NSURG* neurosurgery, *PSURG* plastic surgery, *ENT* otolaryngology, *EYE* ophthalmology, *MFSURG* dentistry, oral and maxillofacial surgery

The involvement of different disciplines in surgery for facial palsy is summarized in Table 3 and in Fig. 2. ENT clinics provided most of the surgical procedures, with a significant gap between them and other disciplines (share of all surgeries: 39%; nerve surgery 56%; sling surgery 39%; upper eyelid weights: 55%; blepharoplasty: 37%; lip/mouth reconstructions: 44%; tightening surgeries: 40%). In second place are the ophthalmic clinics (larger share for tarsorrhaphy: 56% and for other reconstruction on eyelids: 63%) and the institutions for dentistry, oral and maxillofacial surgery. This was followed by the clinics for neurosurgery and plastic surgery (together with otolaryngology larger share for dynamic muscle plastic surgery: 27% each). There were significant differences with regard to the surgical techniques used. While in otolaryngology nerve surgery (operation rate: 0.19 ± 0.06) and sling surgery (0.14 ± 0.05) as well as upper eyelid weights (0.14 ± 0.04) dominated, in ophthalmology, where exclusively operations around the eye were performed, mainly tarsorrhaphy (0.15 ± 0.03) followed by other operations on the eyelids (0.12 ± 0.03) were performed. In neurosurgery, sling surgery dominated (0.12 ± 0.05). In plastic surgery and in dentistry, oral and maxillofacial surgery the methods were distributed without dominance.

**Table 3:** Changes in surgery rates for facial palsy over time from 2005–2019 for the different surgical techniques in the various specialist disciplines involved

Surgical technique	Discipline	Estimate	StdErr	p-value	RR	u. 95%-CI	l. 95%-CI
Nerve surgery	NSURG	0.0314	0.0097	0.0012	1.6024	1.2042	2.1323
	PSURG	0.0482	0.0713	0.4993	2.0605	0.2530	16.7795
	ENT	0.0672	0.0062	< 0.0001	2.7394	2.2802	3.2910
	EYE	– 0.0006	270,916.6	1.0000	0.9905	0.0000	NA
	MFSURG	0.1046	0.0459	0.0225	4.8051	1.2472	18.5123
Muscle transfer	NSURG	0.1021	0.0457	0.0256	4.6242	1.2055	17.7380
	PSURG	0.0537	0.0251	0.0326	2.2389	1.0689	4.6896
	ENT	0.1276	0.0455	0.0050	6.7849	1.7830	25.8185
	EYE	– 0.0006	270,916.6	1.0000	0.9905	0.0000	NA
	MFSURG	0.0910	0.0153	< 0.0001	3.9152	2.4937	6.1472
Sling surgery	NSURG	0.2190	0.0610	0.0003	26.7142	4.4505	160.3538
	PSURG	0.0101	0.0940	0.9141	1.1642	0.0734	18.4550
	ENT	0.0605	0.0100	< 0.00001	2.4797	1.8477	3.3278
	EYE	– 0.0006	270,916.6	1.0000	0.9905	0.0000	NA
	MFSURG	0.0531	0.0090	0.0000	2.2185	1.7027	2.8905
Lid weight	NSURG	– 0.0456	0.1046	0.6627	0.5044	0.0233	10.9219
	PSURG	0.0846	0.0517	0.1017	3.5586	0.7782	16.2738
	ENT	0.0479	0.0111	< 0.00001	2.0518	1.4789	2.8468
	EYE	0.0576	0.0119	< 0.00001	2.3740	1.6749	3.3649
	MFSURG	0.0565	0.0117	< 0.00001	2.3330	1.6535	3.2917
Tarsorrhaphy	NSURG	– 0.0361	0.0165	0.0284	0.5821	0.3587	0.9445
	PSURG	0.0345	0.0272	0.2044	1.6776	0.7545	3.7301
	ENT	0.0346	0.0092	0.0002	1.6792	1.2803	2.2023
	EYE	0.0174	0.0056	0.0018	1.2991	1.1020	1.5315
	MFSURG	0.0039	0.0125	0.7566	1.0596	0.7348	1.5280
Blepharoplasty	NSURG	– 0.2555	0.1911	0.1812	0.0216	0.0001	5.9662
	PSURG	0.0230	0.0887	0.7952	1.4123	0.1042	19.1472
	ENT	0.0424	0.0113	0.0002	1.8888	1.3535	2.6356
	EYE	0.0053	0.0080	0.5112	1.0822	0.8550	1.3696
	MFSURG	0.0280	0.0135	0.0381	1.5218	1.0233	2.2632
Eyelid surgery	NSURG	0.2370	0.2693	0.3788	35.0005	0.0127	96,150.2251
	PSURG	0.0465	0.0941	0.6213	2.0078	0.1264	31.8925
	ENT	0.0311	0.0159	0.0508	1.5934	0.9983	2.5431
	EYE	0.0537	0.0067	< 0.00001	2.2394	1.8401	2.7254
	MFSURG	0.0084	0.0332	0.8002	1.1342	0.4278	3.0070
Lip/Mouth surgery	NSURG	0.0246	0.1934	0.8987	1.4468	0.0049	426.2007
	PSURG	– 0.0413	0.0752	0.5828	0.5382	0.0590	4.9079
	ENT	0.0354	0.0097	0.0003	1.7004	1.2774	2.2634
	EYE	– 0.0006	270,916.6	1.0000	0.9905	0.0000	NA
	MFSURG	0.0322	0.0087	0.0002	1.6218	1.2561	2.0941
Rhytidectomy	NSURG	– 2.7015	2.0315	0.1836	0.0000	0.0000	218,621,251.02
	PSURG	– 0.0040	0.0427	0.9255	0.9418	0.2683	3.3060
	ENT	0.0424	0.0122	0.0005	1.8879	1.3173	2.7056
	EYE	0.2632	0.1794	0.1424	51.8073	0.2652	10,120.8966
	MFSURG	– 0.0239	0.0091	0.0088	0.6992	0.5350	0.9139

*NSURG* neurosurgery, *PSURG* plastic surgery, *ENT* otolaryngology, *EYE* ophthalmology, *MFSURG* dentistry, oral and maxillofacial surgery, *estimate* estimate of the effect of time, *StdErr* standard error of the estimator, *RR* relative risk, *l. 95% CI* lower limit of the 95% confidence interval, *o. 95% CI* upper limit of the 95% confidence interval, *NA* not applicable

For nerve surgery, the greatest increase over time was seen in dentistry, maxillofacial surgery (RR = 4.80; CI = 1.25–18.51) and otolaryngology (RR = 2.74; CI = 2.28–3.29). For dynamic muscle plasty, surgery rates increased the most in otolaryngology (RR = 6.79; CI = 1.78–25.82) and neurosurgery (RR = 4.62; CI = 1.21–17.74). This was also true for sling surgery (neurosurgery: RR = 26.71; CI = 4.45–160.35; otolaryngology: RR = 2.48; CI = 1.85–3.33). Upper eyelid weight implantation increased in otolaryngology (RR = 22.05; CI = 1.48–2.85), ophthalmology (RR = 2.37; CI = 1.67–3.37), and dentistry, oral and maxillofacial surgery (RR = 2.33; CI = 1.65–3.29). Increases for tarsorrhaphy were observed to a lesser extent than for upper eyelid weights in otolaryngology (RR = 1.68; CI = 1.28–2.20) and ophthalmology (RR = 1.30; CI = 1.10–1.53). An increase in blepharoplasties was seen only in otolaryngology (RR = 1.89; CI = 1.35–2.64) and in dentistry, maxillofacial surgery (RR = 1.52; CI = 1.02–2.26). In ophthalmology, reconstructions to the eyelids increased (RR = 2.24; CI = 1.84–2.73). Reconstructions to the lip and mouth increased only in otolaryngology (RR = 1.70; CI = 1.28–2.26) and in dentistry, oral and maxillofacial surgery (RR = 1.62; CI = 1.26–2.09). Rhytidectomy increased significantly only in otolaryngology (RR = 1.89; CI = 1.32–2.71), whereas there was actually a decrease in dentistry, maxillofacial surgery, and oral and maxillofacial surgery (RR = 0.70; CI = 0.54–0.91).

## Discussion

Only for the most common form of acute facial nerve palsy, idiopathic facial nerve palsy, there are comprehensible estimates of the incidence with 20–40 new palsy cases per 100,000 persons per year [8]. About a quarter of all facial palsies have an identifiable cause [9]. This suggests an overall incidence of about 25–55 peripheral facial nerve palsies per 100,000 person-years. Prognosis varies widely, with cure rates ranging from 60 to 80% for idiopathic paresis to 0% for traumatic nerve transection or tumor infiltration [9, 10]. In the more severe lesions, there is mostly defect healing with synkinesis. These patients are predominantly treated conservatively [11]. The incidence of chronic flaccid paralysis developing from acute paresis (including the few cases developing from central paresis, here almost all patients develop at least defect healing) is estimated to be 1–5 per 100,000 person-years. In the present analysis, the mean treatment rate was 2.33 ± 0.53 operations per 100,000 person-years. It is not possible to say how many patients received these operations. It must be assumed that many patients in Germany receive not one but several surgical procedures. This means that the incidences from the patients' point of view are smaller than the surgery rates. Thus, this indicates underuse in Germany, although the increase in surgery rates over time is very welcome. Only a proportion of patients with chronic flaccid facial paralysis seem to receive surgical therapy. On the other hand, it has been proven that patients benefit from facial surgery in terms of function and improvement of quality of life [12–16]. In the United Kingdom, only a small proportion of patients with idiopathic paresis and long-term symptoms are referred to the specialist by the general practitioner (who treats a large proportion of patients in the acute phase in the United Kingdom) [8, 17]. The available data do not allow conclusions to be drawn about hospital referral behavior for Germany. This would require linkage with health insurance data, which is currently impossible using the anonymized DRG data. Actually, it is clear either immediately (tumor, trauma as cause) or at the latest after 6–9 months after idiopathic paresis that a patient cannot show regeneration of the facial nerve or will suffer chronic flaccid paralysis. Nevertheless, at present, not only in Germany, it sometimes takes years until patients are referred to a specialized center [15, 18].

There is not one surgical technique that alone can solve all of the patient's problems. If possible, the facial nerve itself should be reconstructed with a nerve surgery, as this gives the best functional results [2]. The increase in surgical rates over time supports the use of nerve surgery in patients who are candidates for it. Sling surgery are also being used more often, which suggests that more sling surgery (alone or in combination with other procedures) is being used in the first place. Sling surgery has the advantage that leads to immediate functional improvement. Why the increase in surgery rates here, as well as for other techniques, is less in women than in men cannot be explained from the data. Facial paresis is equally common in men and women [1]. A comparison with other countries is also not possible because no such country-wide studies are available internationally. The slight increase in muscle transfer is certainly due to the increased use of free muscle transfer also in adult patients with acquired facial nerve palsies [16]. Nerve surgery or muscle transfer are often combined with measures for ocular closure [2]. Over the years, the use of upper eyelid weight bearing has increased slightly [13]. The use of upper eyelid weighting has increased slightly over the years, especially in ENT clinics, whereas ophthalmic clinics have continued to rely on tarsorrhaphy.

According to the present analysis, the largest surgical spectrum is offered in ENT clinics, and the largest spectrum for surgery around the eye by eye clinics. Surgical rates in the other disciplines were lower, although it should be borne in mind that there are more than twice as many ENT physicians and ophthalmologists in Germany than neurosurgeons and maxillofacial surgeons, and another half as many plastic surgeons. The analysis does not allow us to determine the extent to which patients received interdisciplinary surgical treatment. Treatment in a specialized facial nerve center offers patients a comprehensive interdisciplinary surgical spectrum from a single source [15].

The study has the typical limitations of a DRG analysis that must be considered when interpreting the data. As mentioned above, individual hospital stays and surgeries are considered, but not individual patients. Whether the surgeries were performed during one or multiple inpatient stays cannot be distinguished. In addition, DRG data are originally collected for the purpose of reimbursing a hospital service. Therefore, coding bias due to economic motives cannot be excluded. In particular, adjuvant measures around the eye can also be performed on an outpatient basis. In addition, only coding with ICD code G51.0 could be evaluated, i.e., patients with facial paresis coded with another ICD code could not be considered. Therefore, the surgery rates reported here tend to underestimate the actual number of outpatient and inpatient surgeries. Due to low case numbers, the OPS codes with the first four digits had to be used. Therefore, the different techniques of nerve surgery using different donor nerves, but also the local muscle transfer could not be differentiated from the free muscle transfer which is more important today [16]. In addition, even the 5-digit coding does not allow differentiation of a nerves surgery with an interposition from an end-to-end facial nerve suture, nor does it allow differentiation of the obsolete hypoglossal facial cross nerve suture from the hypoglossal facial jump nerve suture [14]. Thus, DRG statistics are not suitable to analyze in detail whether more current techniques were used over time and obsolete techniques were abandoned for them. Finally, the functional outcome of facial surgery could not be studied. Again, this would require linkage with other hospital data and health insurance data.

## Conclusions

The results make it possible for the first time to take a close look at surgical treatment for facial palsy. Facial surgery has increased significantly from 2005 to 2019, although there is still likely to be an underuse: Even more patients could likely benefit from surgery. Why there are differences between women and men cannot be explained from the data.

## Data Availability

The datasets generated during and/or analysed during the current study are available from the corresponding author on reasonable request.
